# Biological and immunological significance of DLL3 expression in different tumor tissues: a pan-cancer analysis

**DOI:** 10.18632/aging.204672

**Published:** 2023-04-22

**Authors:** Yi Zhang, Lifeng Shang, Junwei Han, Xin Shen, Haiwang Liu, Jie Yang, Hai Shi

**Affiliations:** 1 Department of Gastrointestinal Surgery, Xi’an Daxing Hospital, Xi’an 710000, P.R. China

**Keywords:** DLL3, pan-cancer, immunity, prognosis, microsatellite instability

## Abstract

Objective: To evaluate the biological and immunological significance of DLL3 expression in different tumor tissues and provide insight into the role of DLL3 in tumor immunotherapy.

Material and Methods: RNA expression and clinical data of The Cancer Genome Atlas (TCGA) and Genotype-Tissue Expression (GTEx) were acquired, and we employed couple of bioinformatics methods to investigate the potential biological and immunological role DLL3, including pan-cancer expression, survival analysis, GSVA and it’s correlation with immune infiltration scores, tumor mutation burden, tumor microsatellite instability.

Results: The findings indicate that DLL3 is expressed in the majority of tumors but is only weakly prevalent in HNSC. In 18 different types of cancers, DLL3 expression was linked to TMB and MSI, whereas in KIRC, LIHC, and PAAD, DLL3 expression and TME were correlated. Additionally, DLL3 gene expression linked positively with M0 and M2 macrophage infiltration levels but negatively with the infiltration of most immune cells. And connection with DLL3 expression varied depending on the kind of T cell. Finally, the GSVA data suggested that DLL3 expression is often unfavorably correlated with most pathways.

Conclusions: DLL3 can be used as a stand-alone prognostic factor for many tumor types, and that the level of its expression will have a different prognostic impact for various tumor types. DLL3 expression across numerous cancer types was related to TMB, MSI, and immune cell infiltration. The role of DLL3 in carcinogenesis may serve as a guide for the creation of future immunotherapies that are more individualized and precise.

## INTRODUCTION

Cancers account for the predominant cause of death worldwide, with the dismal 5-year overall survival rates in the world. And the current therapeutic strategies are no definitive [1]. According to the recorded from Europe, about 3.91 million new cases of cancer and 1.93 million cancer-related deaths were recorded in 2018 [2]. Nearly half of all cancer cases in Europe are caused by lung, colorectal, prostate, and female breast cancers collectively [3]. In the last decade, cancer immunotherapy has gained popularity as a cancer therapy, particularly immune checkpoint blocking therapy [4]. By conducting a pan-cancer expression analysis of genes and analyzing their correlations with clinical prognosis and related signaling pathways, it is possible to find new immunotherapy targets owing to the ongoing development and improvement of public databases like The Cancer Genome Atlas (TCGA). Owing to the ongoing development and improvement of public databases like The Cancer Genome Atlas (TCGA), it is now possible to identify new immunotherapy targets by performing a pan-cancer analysis of genes and analyzing their correlations with clinical prognosis and associated signaling pathways [5].

Depending on the cellular context, the single transmembrane protein known as Delta-Like Ligand 3 (DLL3) mediates cell fate decisions and is either tumor-suppressive or carcinogenic [6]. DLL3 is an inhibitory notch ligand that is occasionally expressed in normal tissues. However, several studies showed that DLL3 is highly expressed in small cell lung carcinoma (SCLC) and other neuroendocrine cells neoplasm, which offers potential for DLL3-targeted therapeutic design [7, 8]. Numerous studies have emerged in recent years that demonstrate DLL3’s capacity to bind to several notch receptors and its diverse functions in cell division, proliferation, and apoptosis [9]. DLL3 can have effects that are either pro- or anti-cancer, as mentioned previously. DLL3 has been shown to have anticarcinogenic effects in glioma, hepatocellular carcinoma and malignant glioma, but procarcinogenic effects in SCLC, pituitary tumors, and acute myeloid leukemia. Additional research has revealed that DLL3 performs a range of tasks in cancers by expressing itself in an abnormal manner [10, 11].

Although various researchers have shed light on the function of DLL3 in tumors, their descriptions have only been applied to a certain types of cancer. The relationship between DLL3 and different malignancies has not yet been investigated in a pan-cancer analysis [5–7]. Therefore, in order to examine DLL3 expression levels and their association with prognosis in various malignancies, we utilized a variety of databases, including The Cancer Genome Atlas (TCGA) and Genotype-Tissue Expression (GTEx). We also looked into possible connections between DLL3 expression and levels of immune infiltration, tumor mutational burden (TMB), and microsatellite instability (MSI) in 34 different cancer types. In addition, we performed co-expression analyses of immune-related and mismatch repair (MMR) genes with DLL3 to further investigate the biological roles of DLL3 in malignancies. Overall, this study aimed to evaluate the biological and immunological importance of DLL3 expression in different types of tumor tissues, and provides insight into the role of DLL3 in tumor immunotherapy.

## MATERIALS AND METHODS

### Data collection

Transcriptome, mutation and related clinical data were extracted from UCSC Xena database [12].

### Biological significance of DLL3 expression in tumors

The biological roles of DLL3 in malignancies were examined using GSVA. From the official GSEA website (https://www.gsea-msigdb.org/gsea/downloads.jsp), the Hallmark, KEGG, and GO gene sets were downloaded. The R-packages “GSVA (1.44)”, “org.Hs.eg.db (3.15)”, and “clusterProfiler (4.4)” were used to conduct the functional analysis. The 15 pathways with the most significant associations were at most depicted.

### Relationship between DLL3 expression and immunity

The ESTIMATE algorithm was used to calculate immune and stromal scores infiltrated in tumor samples using R packages “estimate”. And then the relationships between DLL3 expression and those scores were evaluated according to Spearman’s coefficient. Moreover, the metagene tool CIBERSORT was utilized to calculate the relative scores of 22 infiltrated immune cells, and the p value was used for the deconvolution. We also investigated the relationship between DLL3 and immune-related genes, such as those that encode the MHC, immunological activation, immunosuppression, chemokine, and chemokine receptor.

### Analysis of the relationships between DLL3 and prognosis

Survival data were obtained for each sample downloaded from TCGA. To investigate the connection between DLL3 expression and patient prognosis, four indicators: overall survival (OS), disease-specific survival (DSS), disease-free interval (DFI), and progression-free interval (PFI), were chosen. Maximally Selected Rank was utilized to find out the best cut-off to separate the DLL3 expression to higher and lower levels, which could be further analyzed by log-rank test [13]. Cox analysis was conducted using the R packages “survival (3.5)” and “ggplot2 (3.4)” to determine the pan-cancer relationship between DLL3 expression and survival; p < 0.05 were considered significant.

### Correlation of DLL3 expression with TMB, TMI, and MRG expression

TMB scores were calculated based on the total number of gene somatic mutations and base substitutions identified per million bases. The correlations between somatic mutation TMB, MSI and DLL3 expression were examined using Spearman’s coefficient, and the results were shown as the heatmaps by “pheatmap (1.0)” R-package. Cells have a DNA repair system called MMR. Higher frequency of somatic mutations is caused by DNA replication errors that cannot be corrected caused by down-regulation or functional abnormalities in MMR genes. The associations between DLL3 expression and MMR genes were demonstrated using the transcriptomic landscape of pan-cancer from the TCGA.

## RESULTS

### Differential expression of DLL3 between tumor and normal tissue samples

**Figure 1:**
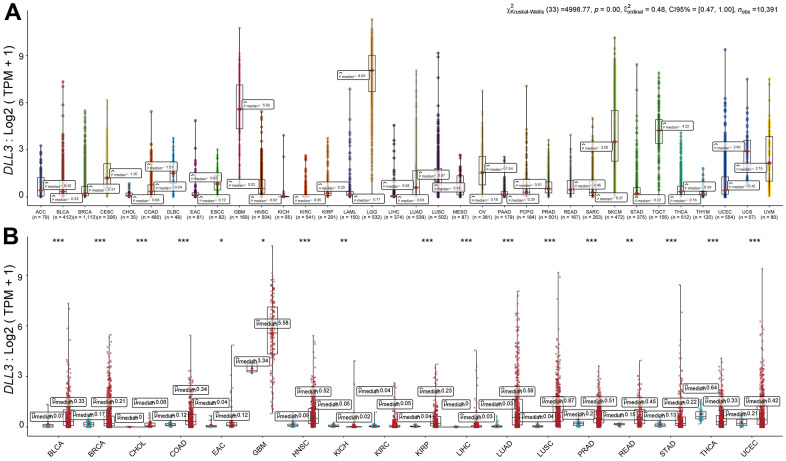
**Differential expression of DLL3.** (**A**) DLL3 expression in 34 types of cancer. (**B**) Comparison of DLL3 expression between tumor and normal samples. *P < 0.05, **P < 0.01, ***P < 0.001.

We examined the levels of DLL3 expression in various malignancies (Figure 1A). DLL3 expression was present in all malignancies, with HGG expressing the most of it and HNSC the least. Based on TCGA data, we also contrasted DLL3 expression levels between matched normal and malignant samples from 34 malignancies (Figure 1B). Significant changes in DLL3 expression between tumor and normal tissue were found in 18 different forms of cancer, with the exception of those where there are fewer than five available normal tissues. Among them, bladder urothelial carcinoma, cholangiocarcinoma, breast invasive carcinoma, COAD, EAC, KIRP, LUSC, PRAD, GBM, HINSC, LIHC, STAD, and UCEC all showed significant levels of DLL3 expression. On the other hands, DLL3 levels were downregulated in thyroid carcinoma and KICH tumors compared to normal tissues (THCA). However, there was no discernible variation in DLL3 levels between KIRC and non-tumor tissues. Of note, the highest difference in DLL3 expression in cancer and normal tissues was for GBM.

### Prognostic value of DLL3 across cancers

**Figure 2:**
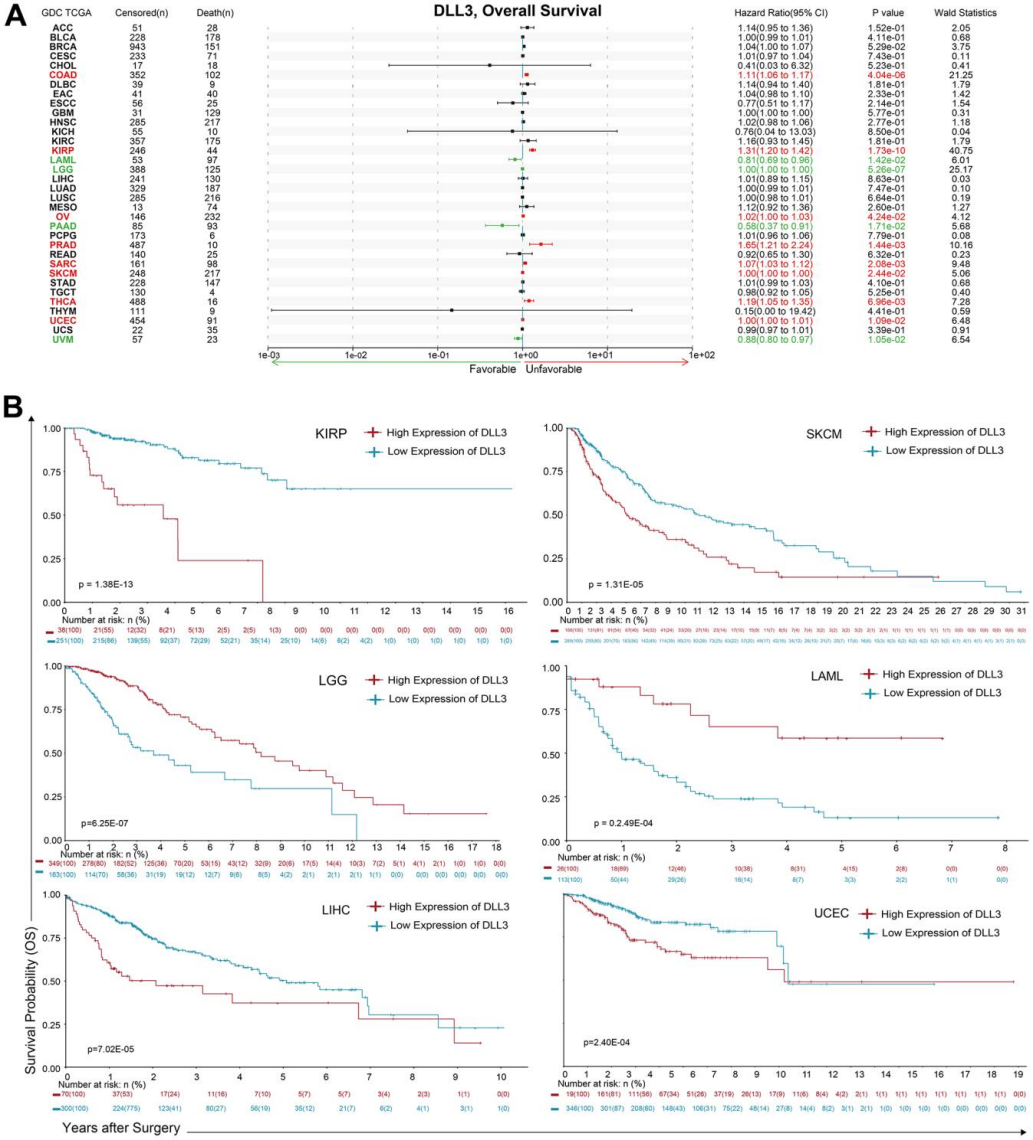
**Association between DLL3 expression and overall survival time in days (OS).** (**A**) Forest plot of OS associations in 34 types of tumor. (**B**) Kaplan-Meier analysis of the association between DLL3 expression and OS.

Cox proportional hazards model analysis revealed that DLL3 expression levels were associated with OS in KIRP (*p* = 1.73e-10), LGG (*p* = 5.26e-07), COAD (*p* = 4.04e-06), PRAD (*p* = 1.44-3e), SARC (*p* = 2.08-3e), THCA (*p* = 6.96-3e), UVM (*p* = 1.05-2e), UCEC (*p* = 1.09-2e), LAML (*p* = 1.42-2e), PAAD (*p* = 1.71-2e), SKCM (*p* = 2.44-2e) and OV (*p* = 4.24-2e) (Figure 2A). Further, DLL3 was a low-risk gene in LGG, THCA, UVM, LAML and PAAD, while it was a high-risk gene in other types of cancer. KM survival analysis demonstrated that there were statistical significances in 23 types of cancer (Supplementary File 1) and the 6 types with dominant significances were shown in (Figure 2B), that is, among patients with LGG (*p* = 2.44e-10) and LAML (*p* = 2.49e-04), those with high levels of DLL3 had longer survival times, while in patients with KIRP (*p* = 1.38e-13), SKCM (*p* = 1.31e-05), LIHC (*p* = 7.02e-05) and UCEC (*p* = 2.40e-04), high DLL3 expression was associated with poor OS.

**Figure 3:**
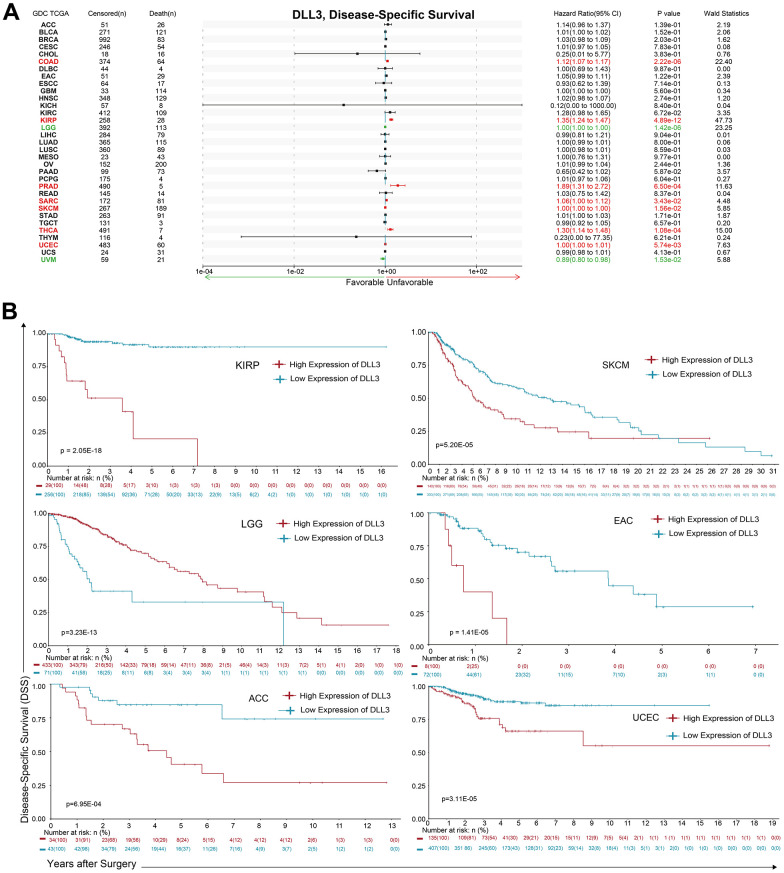
**Association between DLL3 expression and overall survival time in days (DSS).** (**A**) Forest plot of DSS associations in 34 types of tumor. (**B**) Kaplan-Meier analysis of the association between DLL3 expression and DSS.

Moreover, analysis of DSS data (Figure 3A) revealed associations between low DLL3 expression and poor prognosis in patients with LGG (*p* = 1.42e-06) and UVM (p=0.0153); however, in patients with KIRP (*p* = 4.89e-12), COAD (*p* = 2.22e-06), THCA (*p* = 1.08e-04), PRAD (*p* = 6.50e-04), UCEC (*p* = 5.73-3e), SKCM (*p* = 1.56-2e) and SARC (p = 3.43-2e), DLL3 expression exhibited the opposite relationship with prognosis. KM survival analysis presented a positive correlation between DLL3 expression level and poor prognosis in patients with KIRP (*p* = 2.05e-18), EAC (*p* = 1.41e-05), UCEC (*p* = 3.11e-05), SKCM (*p* = 5.20e-05), ACC (*p* = 6.95E-04), except LGG (*p* = 3.23e-13) (Figure 3B). The significance in other 17 types of cancers were shown in Supplementary File 1.

**Figure 4:**
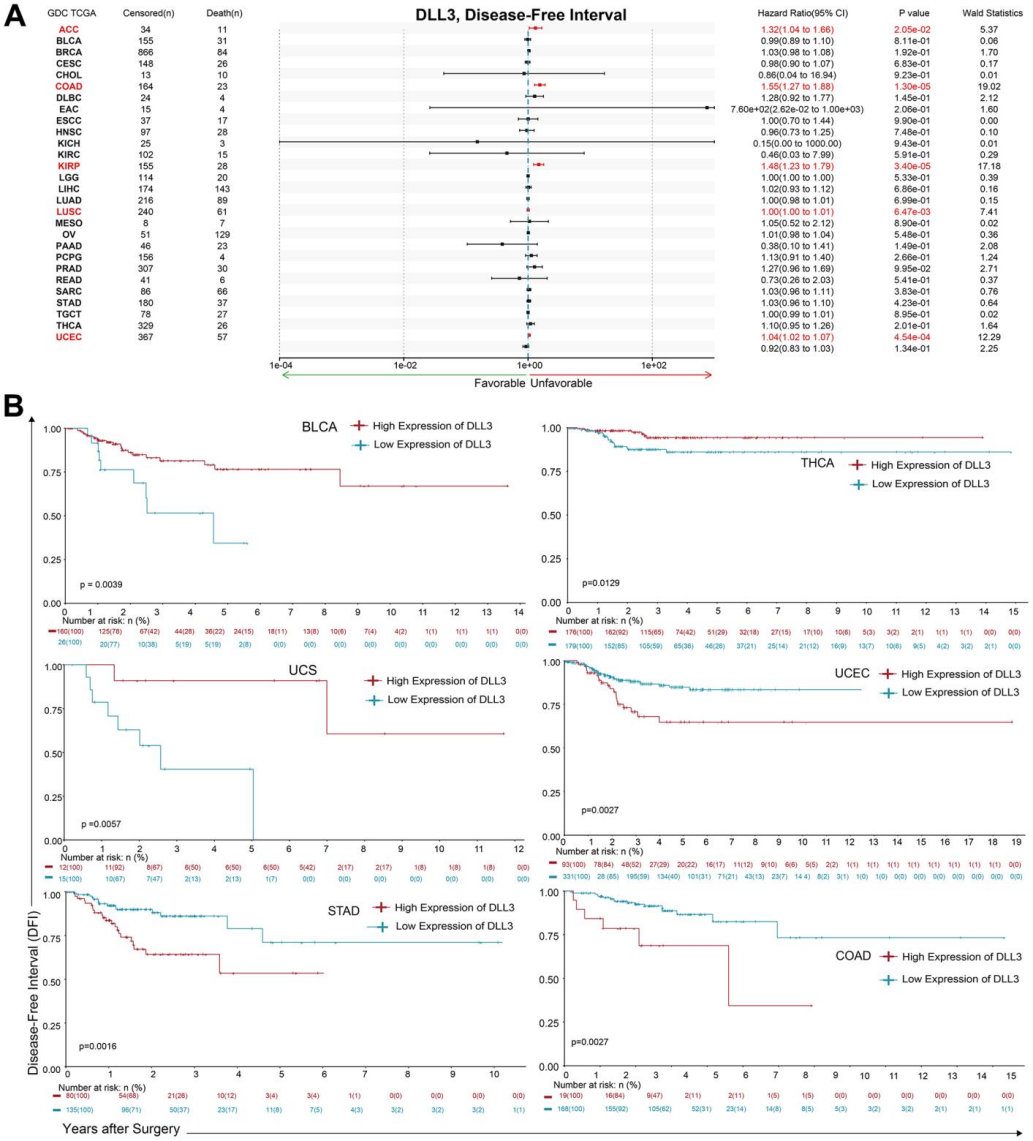
**Association between DLL3 expression and overall survival time in days (DFI).** (**A**) Forest plot of DFI associations in 34 types of tumor. (**B**) Kaplan-Meier analysis of the association between DLL3 expression and DFI.

Additionally, DLL3 expression levels positively correlated with DFI (Figure 4A) in patients with COAD (*p* = 1.30e-05), KIRP (*p* = 3.40e-05), UCEC (*p* = 4.54e-04), LUSC (*p* = 6.47-3e) and ACC (*p* = 2.05-2e). No negative correlation was detected between DLL3 expression and DFI in any type of cancer; however, significant relationships were detected in 16 types of cancers (Supplementary File 1) by KM survival analysis, where the six top significances were observed in Figure 4B. Among patients with UCEC (*p* = 1.98-3e), BLCA (*p* = 3.91-3e), UCS (*p* = 5.65-3e) and THCA (*p* = 1.29-2e), low expression of DLL3 could result in poor prognosis. But there was opposite relationship in STAD (*p* = 1.59-3e) and COAD (*p* = 2.69-3e).

**Figure 5:**
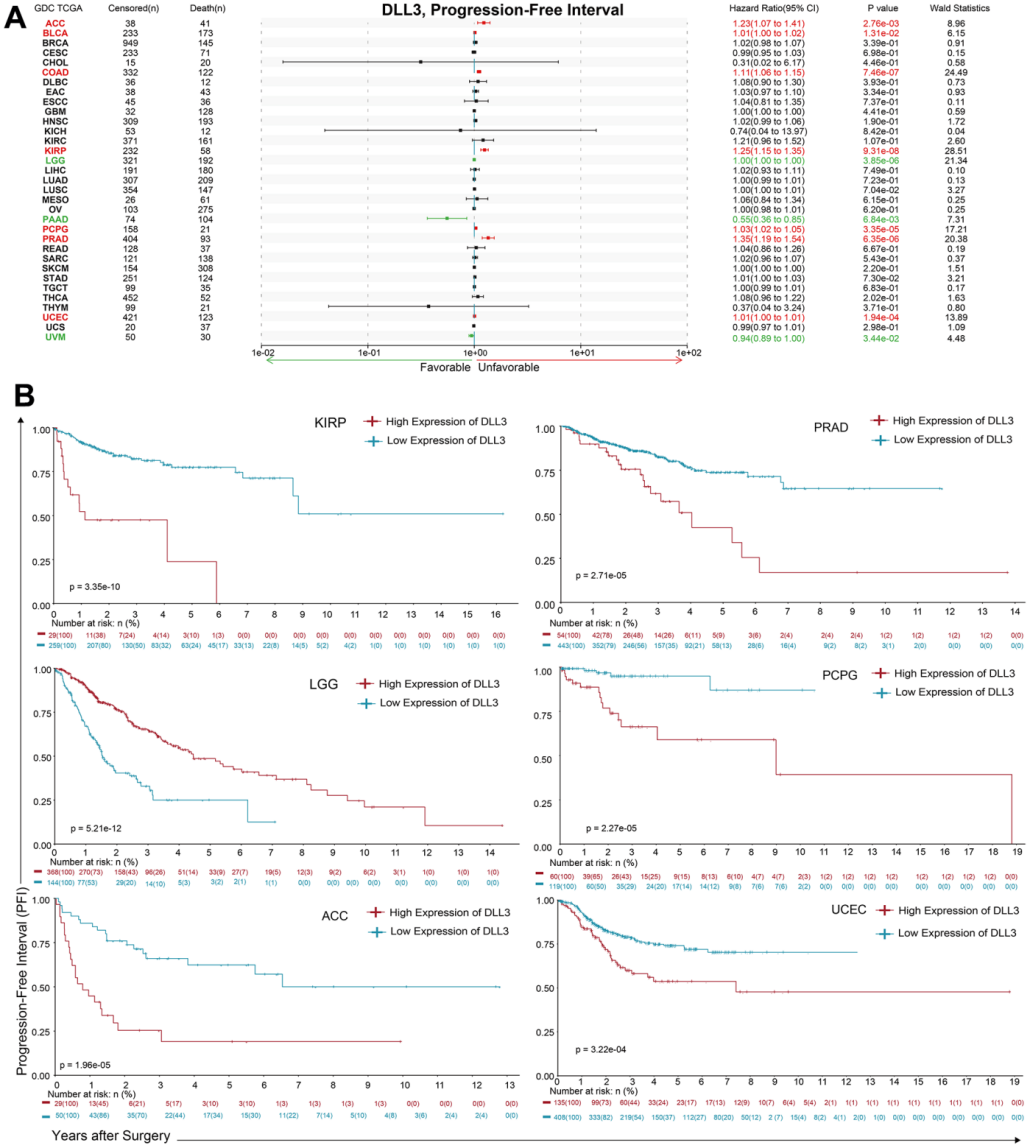
**Association between DLL3 expression and overall survival time in days (PFI).** (**A**) Forest plot of PFI associations in 34 types of tumor. (**B**) Kaplan-Meier analysis of the association between DLL3 expression and PFI.

Regarding associations between DLL3 expression and PFI, forest plots showed associations between high expression and poor PFI in KIRP (*p* = 9.31e-08), COAD (*p* = 7.46e-07), PRAD (*p* = 6.35e-06), PCPG (*p* = 3.35e-05), UCEC (*p* = 1.94e-04), ACC (p = 2.76-3e) and PAAD (p = 6.84-3e), while low expression was associated with poor PFI in patients with LGG (*p* = 3.85e-06), BLCA (*p* = 0.0131) and UVM (*p* = 3.44-2e) (Figure 5A). KM analysis showed that there were statistical significances in 24 cancer types (Figure 5B and Supplementary File 1). individuals with in LGG (*p* = 5.21e-12) and high levels of DLL3 expression had longer survival times, while patients with KIRP (*p* = 3.35e-10), ACC (*p* = 1.96e-05), PCPG (*p* = 2.27e-05), PRAD (*p* = 2.71e-05) and UCEC (*p* = 3.22e-04) and high DLL3 expression had poor PFI.

### The biological significance of DLL3 within tumor microenvironment

**Figure 6:**
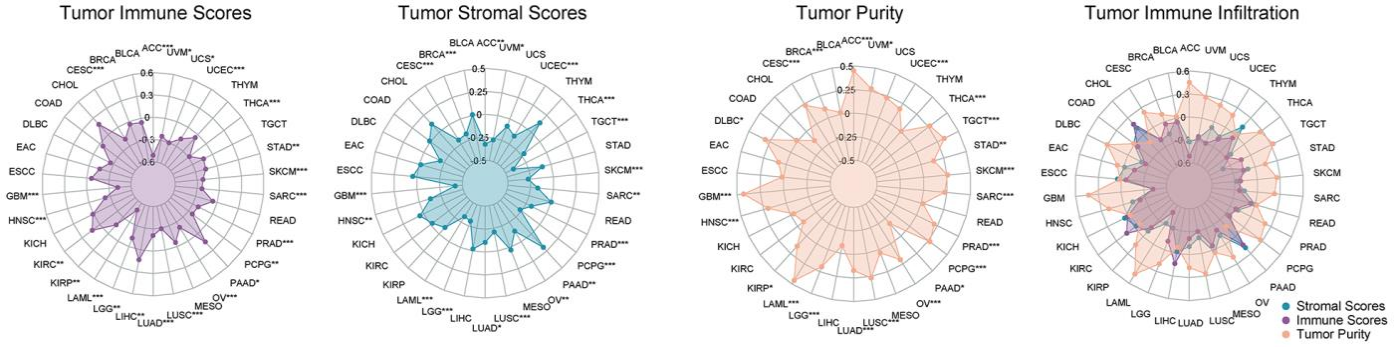
**The correlation coefficients between DLL3 expression and the tumor microenvironment.** Correlation between DLL3 and the immune, stromal, and tumor purity scores in 34 types of cancer.

The microenvironment within cancer entities especially the solid tumors have been indicated play a pivotal role in cancer progression and the immune therapy. We utilized the ESTIMATE algorithm to dissect the non-tumor components, stromal and immune parts, so as to delineate the function of DLL3 in tumor microenvironments and the biological tole in cancer immune therapies. Our results showed that in the pan-cancer study of the KIRC, the expression of LIHC and PAAD was substantially linked with immune scores as well as with stromal scores (Figure 6). Figure 6 also displays the tumors with negative correlation coefficients.

### The biological significance of DLL3 with tumor infiltrating immune cells

**Figure 7:**
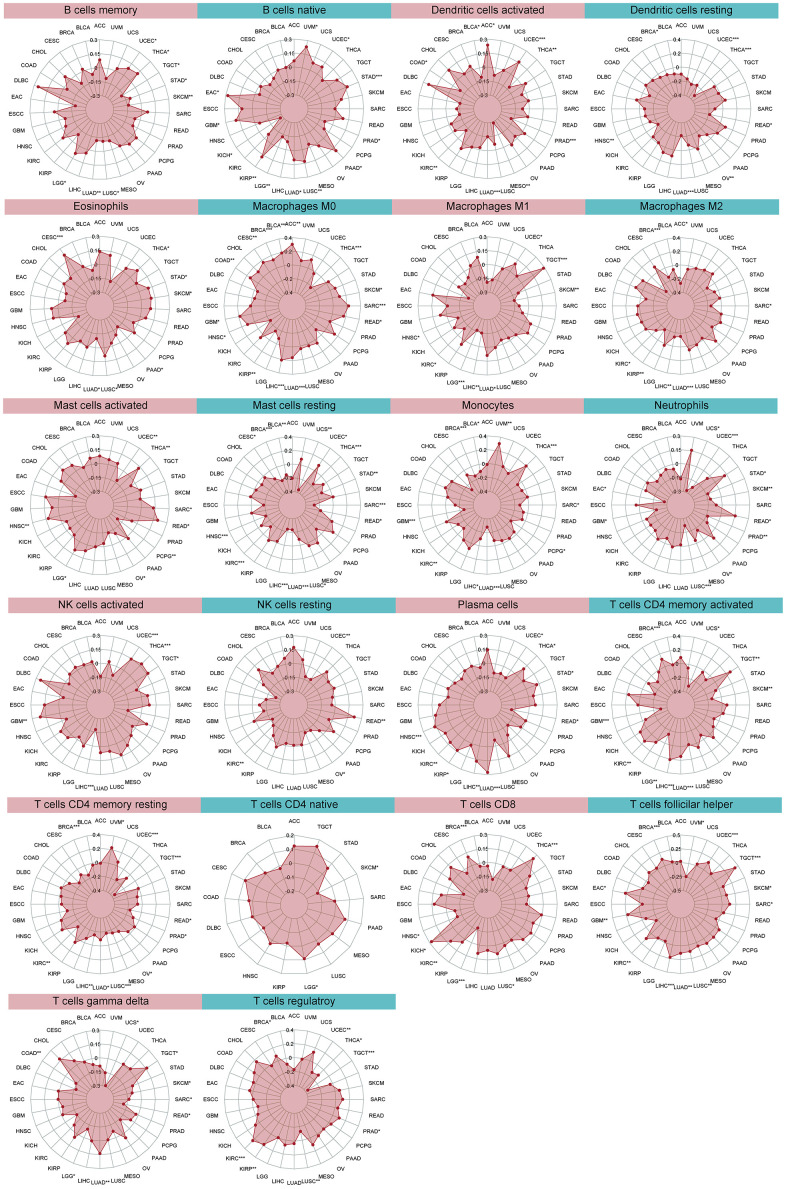
Relationship between DLL3 expression and tumor infiltration of different immune cells.

The relationship between DLL3 expression levels and the tumor infiltrating immune cells were then examined (Figure 7). Our results demonstrate that, in the vast majority of cancer types, levels of immune cell infiltration were significantly linked with DLL3 expression (Supplementary File 2). Six tumors, including BRCA (n = 10), KIRC (n = 12), LIHC (n = 10), LUAD (n = 15), THCA (n = 12), and UCEC (n = 14), with the strongest correlations between DLL3 expression and immune cell infiltration underwent further examination.

In the six malignancies examined, DLL3 expression levels were negatively linked with dendritic cell, macrophage, mast cell, and T cell infiltration rates. DLL3 expression levels were also linked to numerous distinct subgroups of engulfing macrophages. For instance, DLL3 expression showed a positive correlation with invading M0 macrophage levels in LIHC but a negative correlation with infiltrating M1 macrophage levels. Similar to this, DLL3 expression and levels of invading M2 macrophages were positively correlated with LUAD, BRCA, LIHC, and KIRC.

Additionally, several relationships between DLL3 expression levels and various subsets of tumor-infiltrating T cells were found. Except in THCA, DLL3 expression was inversely connected with levels of invading resting CD4 memory T cells, but positively correlated with levels of follicular helper T cells. In addition, we found a negative connection between these six tumors, with the exception of UCEC, and the quantity of resting mast cells. Figure 7 shows the association between DLL3 expression and the invasion of various immune cells into tumors.

**Figure 8:**
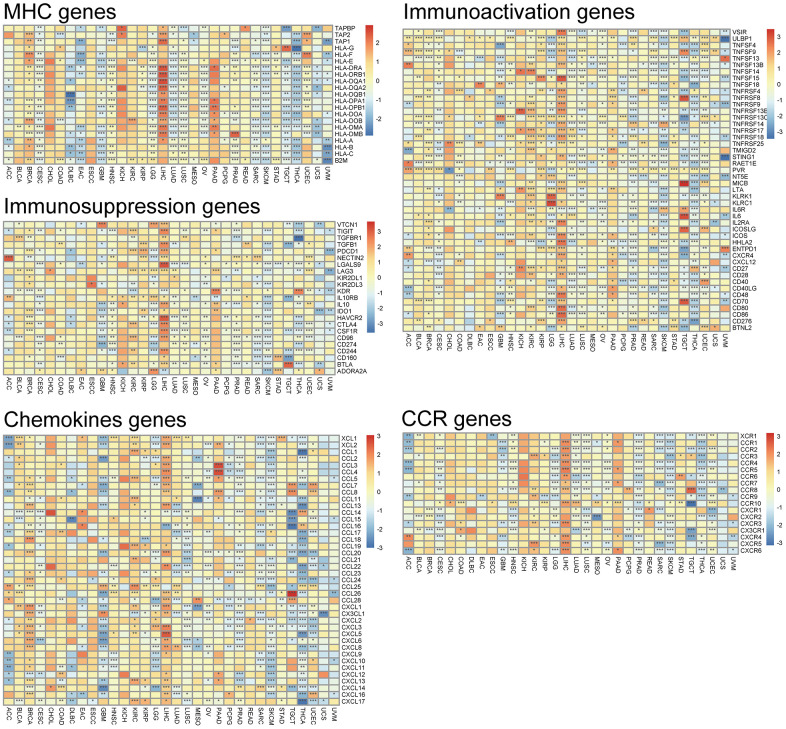
**Co-expression of DLL3 and immune-related genes.** *P < 0.05, **P < 0.01, ***P < 0.001.

Almost all immune-related genes presented the co-expressed pattern with DLL3 expression (Figure 8), and the most of them were positively correlated with DLL3 in all kinds of tumor except for several types of tumors (SARC, SKCM, TGCT, UVM, and THCA).

### The potential role of DLL3 within TMB and MSI

**Figure 9:**
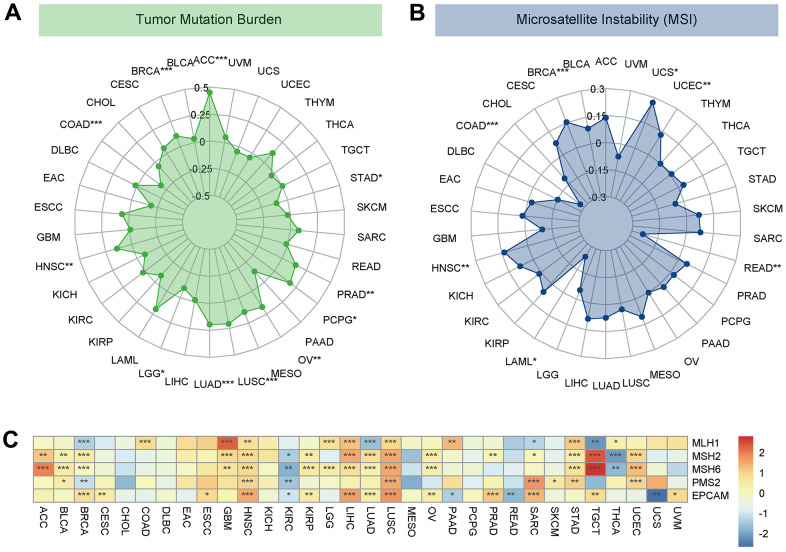
**Associations between DLL3 expression and tumor mutational burden (TMB), microsatellite instability (MSI), and mismatch repair (MMR).** (**A**) Radar plot illustrating the relationship between DLL3 and TMB. (**B**) Radar plot illustrating the relationship between DLL3 and MSI. (**C**) Heatmap illustrating the association between DLL3 expression and MMR genes. *P < 0.05, **P < 0.01, ***P < 0.001.

Then, because TMB and MSI are crucially related to the sensitivity of immune checkpoint inhibitors, we looked into whether DLL3 expression levels and those variables were correlated. Consequently, we investigated the connections between the levels of MMR genes and DLL3. The results demonstrated that, in 11 types of tumors, *e.g.*, ACC, BRCA, COAD and LUAD, LUSC, DLL3 expression was related to TMB (Figure 9A). In another 7 types of tumors, including BRCA, COAD, HNSC, LAML, READ, UCEC and UCS, DLL3 expression was related to MSI (Figure 9B). Moreover, MMR gene expression was clearly and significantly positively correlated with DDL3 levels, especially in HNSC, LUSC and KIRC. But negative correlation was displayed in a few tumors, such as KIRC (Figure 9C).

### Pan-cancer biological exploration of DLL3

**Figure 10:**
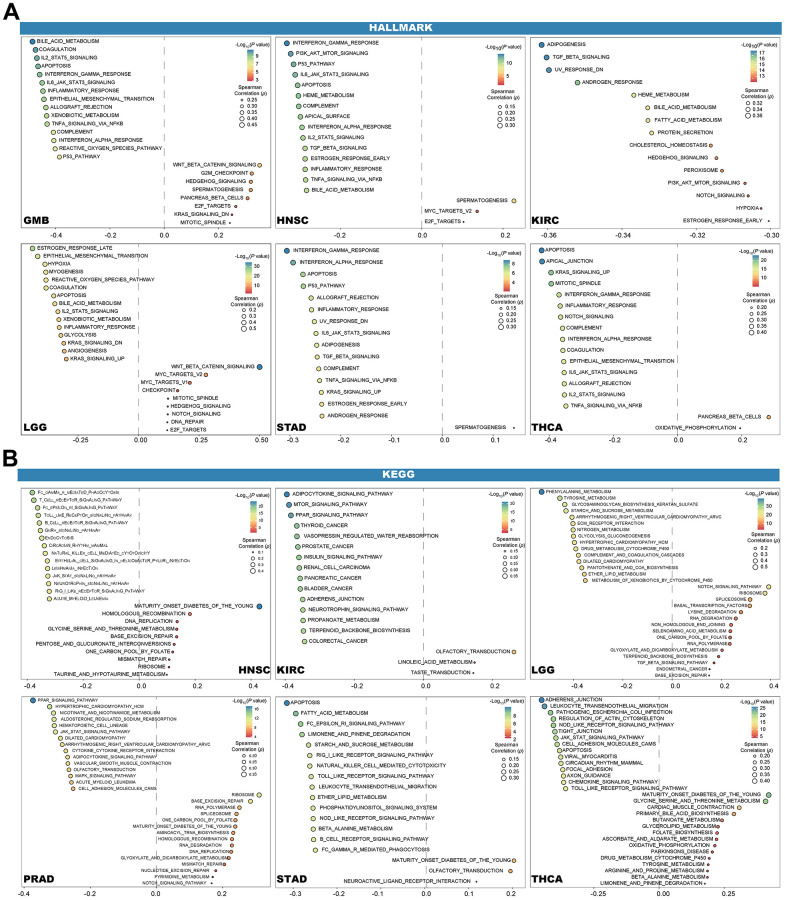
**Results of GSVA.** Bubble plot show the 15 pathways of Hallmark (**A**) and KEGG (**B**) with the most significant positive and negative, respectively.

GSVA was utilized to further explore the biological significance of DLL3 expression in six tumors, including GBM, HNSC, KIRC, LGG, STAD and THCA. The top 15 pathways of hallmarks that are significantly positively and negatively associated with DLL3 expression in each tumor are presented in Figure 10A. The results demonstrate that DLL3 expression is negatively associated with most pathways, with exception of spermatogenesis, E2F targets and MYC targets.

Additionally, another six tumors with the largest number of most significant pathways were shown in Figure 10B, of which KIRC, STAD and THCA were shared by hallmarks and KEGG. Similar to the results in hallmarks, in the six tumors in KEGG, DLL3 expression negatively correlated with most pathways, while MATURITY_ONSET_DIABETES_OF_THE_YOUNG showed a positive association with HNSC, THCA, PRAD and STAD. Other positive correlations with the same pathway did not occur in more than half of the six tumors.

## DISCUSSION

The notch signaling pathway, which affects a number of cellular functions including differentiation, proliferation, survival, and apoptosis, depends heavily on DLL3 [6, 14]. In this study, we discussed the biological and immunological significance of DLL3 expression in different tumor tissues. Our findings demonstrated that the DLL3 is substantially expressed in 34 different cancer types and has a significant role in tumor immunity by influencing immune cells that infiltrate tumors. According to our study findings, DLL3 affects cell adhesion and a number of immunological-related processes in LGG and KICH.

We demonstrated that the potential oncological role of DLL3 in patients with cervical squamous cancer (CESE). However, previous studies showed that patients with DLBC and high levels of DLL3 expression had longer survival times, while patients with LGG and PRAD and high DLL3 expression had poor PFI [15]. Additionally, previous studies have shown a favorable correlation between the pathological stage of lung cancer and DLL3 expression on lung cells [9]. These results unequivocally show that DLL3 can be utilized as a biomarker to assess the prognosis of different malignancies.

In the era of precision medicine, TMB is a promising pan-cancer prognostic biomarker that can guide immunotherapy [16]. Previous studies have demonstrated that TMB can be utilized to predict the prognosis after immunotherapy in pan-cancer patients [17, 18]. In immune-checkpoint inhibitors (ICI), MSI is a crucial biomarker [18]. High-frequency MSI is an independent predictor of clinical characteristics and prognosis for colorectal cancer. This study showed that DLL3 expression was associated with TMB in 12 different tumor types, including breast, colorectal, lungs, glioma, and kidney cancer. DLL3 expression was associated with MSI in 12 other tumor types, including lymphoma, colorectal, lungs, and stomach malignancies. MMR gene expression was clearly and significantly inversely linked with DLL3 levels in the majority of cancers, with the exception of LIHC. In malignancies where DLL3 is present, tumors with high DLL3 expression, high TMB, and high MSI may have a better prognosis after ICI treatment, according to our results and earlier research.

Our findings demonstrate that DLL3 is crucial for cancer immunity. TME characteristics have an impact on clinical outcomes and are used as indicators to assess tumor cell responses to immunotherapy. According to the results of the ESTIMATE algorithm scores for 34 genes, DLL3 expression was strongly positively connected with immune scores as well as with stromal scores in a pan-cancer analysis, with the exception of DLBC, LAML, and THYM. Immune cells that have invaded tumors have significant effects on the emergence and growth of malignancies and can either oppose or favor tumor emergence and growth. The findings show that DLL3 expression is favorably correlated with a number of immune cell- and immune factor-related processes, including cell migration, synaptic pruning, and B, CD4 T, and CD8 T cells. Subsequent examination of our data showed that DLL3 expression was significantly correlated with immune cell infiltration levels in the majority of cancer types. Thereafter, these malignancies were screened for additional investigation.

In conclusion, our findings imply that DLL3 can be used as a stand-alone prognostic factor for many tumor types, and that the level of its expression will have a different prognostic impact for various tumor types. This suggests that further research is necessary to determine the precise function of DLL3 in each type of cancer. Furthermore, across numerous cancer types, DLL3 expression was related to TMB, MSI, and immune cell infiltration. The influence on tumor immunity varies based on the type of tumor. These findings may shed light on the function of DLL3 in carcinogenesis and development and may serve as a guide for the creation of future immunotherapies that are more individualized and precise.

## Supplementary Material

Supplementary File 1

Supplementary File 2
